# Screening and Targeting Risk Factors for Prodromal Synucleinopathy: Taking Steps toward a Prescriptive Multi-modal Framework

**DOI:** 10.14336/AD.2022.1024

**Published:** 2023-08-01

**Authors:** Lee E Neilson, Joseph F Quinn, Miranda M Lim

**Affiliations:** ^1^Department of Neurology, Veterans Affairs Portland Healthcare System, Portland, OR 97239, USA.; ^2^Department of Neurology, Oregon Health and Science University, Portland, OR 97239, USA; ^3^Department of Behavioral Neuroscience, Oregon Health and Science University, Portland, OR 97239, USA.; ^4^Department of Medicine, Division of Pulmonary and Critical Care Medicine, Oregon Health and Science University, Portland, OR 97239, USA.; ^5^Oregon Institute of Occupational Health Sciences, Oregon Health and Science University, Portland, OR 97239, USA.

**Keywords:** Parkinson’s disease;, neurodegenerative disease, prodromal, intervention, neuroprotection, REM sleep behavior disorder

## Abstract

As the prevalence of Parkinson’s disease (PD) grows, so too does the population at-risk of developing PD, those in the so-called prodromal period. This period can span from those experiencing subtle motor deficits yet not meeting full diagnostic criteria or those with physiologic markers of disease alone. Several disease-modifying therapies have failed to show a neuroprotective effect. A common criticism is that neurodegeneration, even in the early motor stages, has advanced too far for neuro-restoration-based interventions to be effective. Therefore, identifying this early population is essential. Once identified, these patients could then potentially benefit from sweeping lifestyle modifications to alter their disease trajectory. Herein, we review the literature on risk factors for, and prodromal symptoms of, PD with an emphasis on ones which may be modifiable in the earliest possible stages. We propose a process for identifying this population and speculate on some strategies which may modulate disease trajectory. Ultimately, this proposal warrants prospective studies.

## 1. Introduction

Parkinson’s disease (PD), the canonical disease characterized by alpha-synuclein deposition [1], affects 1% of the population over age 60 [2], making it the second most common neurodegenerative disorder [3]. The prevalence of the disease is expected to rise dramatically in the coming decades [4], and consequently its annual cost is projected to rise over 50% to $79 billion by 2037 [5]. While only able to be diagnosed once the motor manifestations of tremor, rigidity, and bradykinesia emerge [6], predictive models based on diagnosis of idiopathic rapid eye-movement (REM) sleep behavior disorder (RBD) and other risk factors make it possible to identify people in the prodromal stage of synucleinopathy who have a high probability of future disease [7]. Once identified, these patients could then potentially benefit from sweeping lifestyle modifications to alter their disease trajectory.

The consequence of proactively disclosing this high-risk status is that many patients may feel at a loss for what next steps can be taken for their health. Traditional rehabilitative therapies for PD are delivered only after diagnosis or even later when physical function is impaired, but by this point, substantial neurodegeneration has already occurred [8] which may limit the effectiveness of any intervention. Understanding this creates a window during which neuroprotective interventions may be proactively applied. Alzheimer’s disease and related dementias (ADRD) have a comprehensive proposal for policy and individual interventions to target 12 key risk factors, and this is projected to yield a 40% reduction in the prevention or delay of onset [9].

The World Health Organization recently identified six challenges to addressing the burden of PD and has placed an urgent call to specifically ‘generate harmonized approaches for PD risk reduction based on existing evidence, with both individual-level and population-level approaches’ [10]. Heretofore, only one proposal has been offered within the RBD population, based solely on exercise [11]. Herein, we offer an initial proposal for a ‘prescription’ for a multimodal approach to delaying disease onset at the level of the individual. This first step is based on extensive literature review and our collective experience with this population. We also summarize some of the negative studies that are important to share with patients as they may be susceptible to misleading marketing claims. Finally, we consider a screening protocol to capture this population-at-risk for a neuroprotection clinic. The authors hope that this publication will serve as a straightforward and convenient guide for all clinicians caring for patients at risk of neurodegenerative parkinsonian disorders.

**Figure 1.**
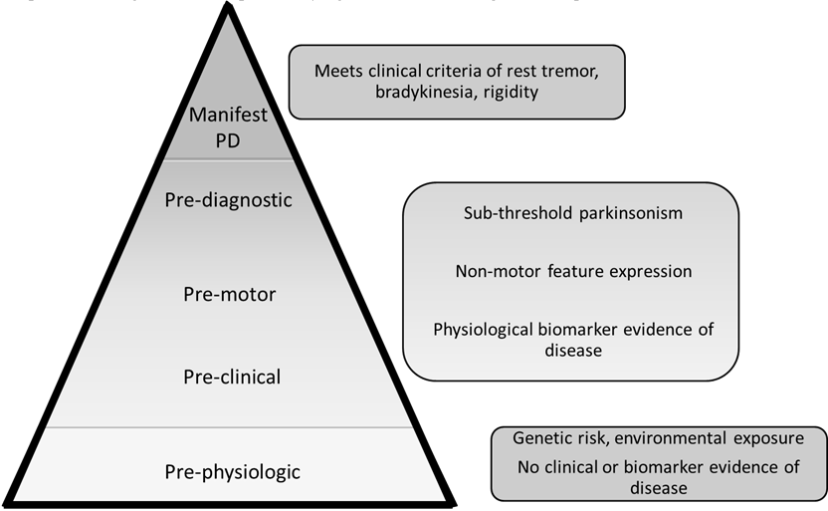
**The Parkinson’s disease At-Risk Syndrome (PARS) pyramid**. In this conceptual schema, four stages precede clinically manifest PD: pre-physiological, pre-clinical, pre-motor and pre-diagnostic. The prodromal period includes the middle categories where there is evidence of disease activity but there is no fulfillment of the PD diagnostic criteria.

## 2. Identifying Prodromal Synucleinopathies

PD, like most neurodegenerative diseases, is an insidious process, which implies the disease progresses through phases. In fact, by the time the disease is diagnosed, moderate loss of dopaminergic neurons within the substantia nigra has already occurred [12]. This process can be captured with the Parkinson’s Disease At Risk Syndrome (PARS) schema, initially proposed by Siderowf and Lang [13] (Fig. 1). At the base is the pre-physiological phase which accounts for the baseline risk in accord with genetic status, environmental exposures, and chronological age that are otherwise immutable. At this point, however, instruments are not sensitive enough to detect any physiological changes consistent with PD. Next is the prodromal period which comprises the pre-clinical, the pre-motor, and the pre-diagnostic phases. In the preclinical stage, the individual may display no signs or symptoms of parkinsonism, but available testing can detect some changes in physiology (e.g., dopaminergic transmission deficit through dopamine transporter scans or alpha-synuclein aggregation through skin biopsies). In the premotor phase, an individual may start to experience some symptoms such as depression or constipation which are clearly linked to the underlying pathologic process of PD, but no motor symptoms are present where the diagnosis can be definitively made. Next is the pre-diagnostic phase where the patient has come to a neurologist’s attention with some motor impairments, perhaps gait dysfunction or a mild tremor, but again the full criteria for PD are not yet met. It is anticipated, however, that they will progress to frank parkinsonism. Finally, the peak of the pyramid is manifest PD.

Since the diagnosis cannot be made without manifest motor symptoms, attempts have been made to identify this prodromal period based on various risk markers and the presence of at least one prodromal marker. Risk markers, or risk factors, are not early manifestations of the disease but rather predispose one to developing the disease. For example, while aging is a strong risk factor, the act of aging does not imply PD is present. In contrast, prodromal markers refer to any indicator, be it sign, symptom, or laboratory biomarker, which indicates neurodegeneration is present [14]. Given that there are currently no means to identify prodromal PD with 100% certainty, diagnostic criteria for prodromal PD are based on probability, with 80% likelihood serving as the cutoff. Due to the rapidly evolving nature of this field, the criteria demand to be updated often [7].

Reliable identification of the prodromal condition could enable early intervention, mitigate iatrogenic adverse events, reduce emergency care utilization, and facilitate advanced care planning while pathologic burden is low and before clinical symptoms become debilitating. Therefore, once identified, we propose selecting prodromal symptoms and risk factors which may be amenable for modification.

## 3a. Non-Modifiable Risk and Prodromal Factors

Several risk factors have been identified for the development of synucleinopathies including chronological age, sex, genetic status, first-degree family history, prior occupational hazards (including pesticide, manganese and Agent Orange exposure), hyposmia, and RBD [7]. In contrast to chronological age, biological age refers to epigenetic modifications to the genome that may further alter the risk of neurodegenerative disease. As an example, hypo-methylation of cytosine/guanine (CpG) dinucleotides has been linked to enhanced alpha-synuclein expression [15, 16]. Some research points to the potential for modification of these changes through diet and exercise [17], and will thus be further explored in the sections below. Lastly, while some targeted therapeutics are currently under investigation for the LRRK2 and GBA mutations (ClinicalTrials.gov Identifiers: NCT03976349 and NCT04127578), these are otherwise considered non-modifiable and thus outside the scope of this review. While non-modifiable, RBD is treatable. After evaluating for environmental safety and eliminating drugs which can worsen symptoms (e.g., selective serotonin reuptake inhibitors, serotonin-norepinephrine reuptake inhibitors, and tricyclic antidepressants), initiation of melatonin should be considered. In addition to its benign side effect profile, it has been shown to improve the REM without atonia on PSG [18]. Several animal studies have demonstrated a cytoprotective, antioxidant, and pro-locomotive effect with melatonin [19] but limited explorations have been undertaken in human trials [20, 21].

## 3b. Modifiable Risk Factors

Risk factors are any co-morbid conditions or behaviors that increase susceptibility to disease. Table 1 summarizes the risk factors for PD, with greater detail offered below.

*Smoking*. Whereas the risk of most non-communicable diseases is higher in smokers than non-smokers, PD is a curious exception. An early meta-analysis of 48 prospective case-control and cohort studies showed that, compared with never smokers, the relative risk of PD was 0.80 for past smokers, and 0.39 for current smokers [22]. Later studies confirmed a causally-protective effect [23] and suggested it to be among the strongest protective factors [24]. The mechanisms underlying the inverse association between smoking and PD are not fully understood, though some studies have implicated nicotine in neuroprotection[25]. In fact, one population-based study has shown that those who consume greater amounts of foods with nicotine-containing compounds (e.g. peppers, eggplants, tomatoes, and potatoes) show a similar protective effect [26]. Yet others have suggested that genetic factors [27] or personality traits[28] may predispose to smoking behavior and modify PD risk. Various animal studies support a neuroprotective role of nicotine [29, 30].. Nicotine patches have been explored in clinical trials without demonstrable benefits in motor symptoms, but significant improvement in some non-motor features suggests further study in larger scale trials may be warranted [31]. Until the truly beneficial compounds in tobacco are isolated and proven safe and protective, given the deleterious effects on general health, smoking should not be advocated.

*Diabetes*. Several large prospective studies in Finnish [32], American [33, 34], Chinese [35], Taiwanese [36] and British [37] adults have shown a link between diabetes mellitus and incident PD, with a pooled positive likelihood ratio (LR+) of 1.5 [7]. While good glycemic control cannot be understood to forestall the development of PD, there are myriad benefits including maintenance of muscle mass and exercise tolerance [38] as well as on preventing retinopathy, peripheral polyneuropathy, and cerebrovascular disease [39], each of which can contribute to gait difficulties. More recently, exenatide, a glucagon-like peptide-1 (GLP1) receptor agonist used in the treatment of diabetes mellitus has shown promise as a neuroprotective agent. A pooled analysis of two randomized, controlled, clinical trials of exenatide indicated some benefits in cognitive test scores, quality of life, and reductions in non-motor symptoms and motor complications in patients with PD [40]. These motor benefits persisted for 12 months. Further studies are ongoing, but a provider may consider prescribing a GLP1 receptor agonist when treatment for euglycemia is indicated in those at high risk of PD.

*Vascular Risk Factors*. Vascular risk factors such as hypertension, hyperlipidemia, tobacco use, hyper-glycemia, and other syndromes which predispose to cerebrovascular disease (e.g., atrial fibrillation) contribute to the breakdown of the neurovascular unit and can drive accumulation of vascular lesions in the brain. This process can drive vascular cognitive impairment [41] and/or vascular parkinsonism [42]. It is not merely the presence of such factors which portend poor outcomes, but the rate of accumulation and changing severity. An accelerated, as opposed to a stable, cardiovascular risk trajectory predicts an increased risk in developing dementia of either the Alzheimer’s or vascular type [43]. Based on this data, the American Heart Association/American Stroke Association (AHA/ASA) supports the “reasonableness” of smoking cessation, weight control, increased physical activity, and treatment of hyperglycemia and hypercholesterolemia. Regarding the latter, pooled data from three ongoing clinico-pathologic studies (the Religious Orders Study, the Rush Memory and Aging Project, and the Minority Aging Research Study) suggest that statin users had a 12% lower risk of incident parkinsonism, even when adjusting for vascular disease and risk factors [44].

The AHA/ASA offers a stronger recommendation to treat hypertension[41]. The choice of antihypertensive remains at the discretion of the internist; however, a few studies are worth considering. One retrospective case-control study of US Medicare beneficiaries indicated a significant risk of developing incident PD (OR = 3.62) with propranolol use [45]. This was not a class effect; other beta-2-adrenergic receptor antagonists did not show a similar association. This propranolol effect was entirely mitigated once correcting for pre-morbid tremor, suggesting these patients may have been in the prodromal phase already. That being said, a more recent study showed the same increased risk with propranolol while also linking the beta-2-adrenergic pathway to alpha-synuclein gene expression [46], suggesting a drug-specific effect. Lastly, a meta-analysis showed that while angiotensin converting enzyme (ACE) inhibitors (ACEI), and angiotensin II receptor blockers (ARBs) did not have an effect on incident PD, calcium channel blockers (CCBs) showed a reduced incidence with a modest effect size [47]. While this observation and other work in animal models were promising, a prospective clinical trial with isradipine, a dihydropyridine CCB, was ultimately negative[48]. Post hoc secondary analyses indicated those with prolonged exposure of a higher dose of isradipine delayed the need for symptomatic therapy [49]. Thus, further studies are ongoing.

*Physical Inactivity*. While early cohort studies offered mixed results, more recently, researchers have shown a consistent inverse relationship between physical activity and PD incidence [7]. A large prospective Finnish study demonstrated that participants with heavy non-occupation physical activity had a lower PD risk than those with no activity [50]. Another group showed a dose-response relationship with exercise and incident PD in a large US prospective cohort study and a corresponding meta-analysis [51].

Endurance exercise has consistently shown a symptomatic benefit in patients with PD in prospective studies [52, 53]. While animal studies have shown a neuroprotective effect [54, 55], no such study has definitively demonstrated the same in humans. One meta-analysis pooled 8 prospective studies with a total of more than 500,000 adults followed up for an average of 12 years, with more than 2,100 PD cases and showed a significantly reduced risk of PD with the highest level of total physical activity compared with the lowest level [56]. A more recent study of 1251 PD patients showed the same robust findings [57]. However, it was only the phase II Study in Parkinson Disease of Exercise (SPARX2) trial which was powered to look at a disease modifying effect [58]. They randomized de novo, untreated patients with PD to high- (80-85% maximum heart rate) or moderate-intensity (60-65% maximum heart rate) regimen for three days weekly. In addition to showing safety and strong adherence to the prescribed regimen, there was a significant reduction in progression of motor outcome measures in the high-intensity group. A phase III efficacy trial (SPARX3) is ongoing to show that this association is not a result of reverse causation, that is, that people with prodromal parkinsonism have subtle manifestations which limit their participation in exercise (clinicaltrials.gov/NCT04284436).

Even if the prescribed exercise regimen from SPARX3 does not show a disease-modifying effect, we would be hard-pressed to abandon exercise as part of the recommendations. Those with frank PD and higher physical activity improved cognitive function, irrespective of APOEε4 allele status [59]. Other interventional studies have shown similar improvements in cognitive outcomes compared to waiting list controls [60, 61]. Lastly, other domains, such as postural stability, can be improved even without attaining high heart rates, such as through Tai Chi [62].

*Caffeine*. Several large retrospective and prospective cohort studies have evaluated the risk of incident PD based on reported caffeine intake. An early study in Japanese-American men showed an inverse dose-response relationship with coffee [63], and a later study confirmed the finding in both men and women for caffeinated beverages of various types [64]. A large prospective study of over 300,000 participants, and a subsequent meta-analysis, corroborated earlier findings that caffeinated beverage consumption - at least 3 cups of coffee (~100mg/cup) or 6 cups of black tea (~50mg/cup) per week - inversely correlated with PD incidence in both men and women when correcting for smoking status, alcohol intake, and physical activity [65]. Caffeine, which is able to cross the blood-brain barrier, has been shown to reduce the production of reactive oxygen species (ROS) and thereby mitigate the loss of dopaminergic neurons, perhaps through the activation of the nuclear factor erythroid 2-related factor 2 (Nrf2) pathway or through blocking adenosine 2A receptors (A2AR) [66]. One drug, istradefylline, is a known A2AR antagonist which has shown a symptomatic, but not a disease-modifying, benefit [67]. Interest has therefore shifted to broader targets. One prospective double-blind, controlled phase 2/3 study of PD investigated a 6-week course of 200mg daily of caffeine and demonstrated improvements in sleepiness and a modest improvement in motor scores [68]. A second study investigated the same 200mg daily dose of caffeine, but over an 18 month period, and recapitulated the improvement in sleepiness but failed to show a significant improvement in motor outcomes [69].

*Diet and Nutritional Supplements*. Dietary research is historically fraught with challenges, but some detailed recent work has shed light on possible associations of dietary choice and incident parkinsonism, with particular emphasis on the Mediterranean diet (MeDi) ever since the observation that Mediterranean populations with adherence to this diet had lower mortality [70]. Grossly, the key beneficial components of the MeDi include vegetables, legumes, fruits and nuts, cereal, fish, a high polyunsaturated: saturated fats ratio, and a moderate daily amount of alcohol intake. A moderate amount is considered to be no greater than 2 standard drinks per day for men, and just 1 for women. The detrimental components include meat, poultry, and dairy products. With 1 point awarded for each component relative to population mean, a MeDi score, and by extension adherence, can be calculated.

Using this method, groups in the US [71] and Sweden [72] have shown an inverse relationship between MeDi adherence and incident PD. Yet other groups have shown that MeDi adherence lowers the risk of probability of prodromal PD in both retrospective cross-sectional [73] and prospective longitudinal [74] studies. A refined version of the MeDi, incorporating some components of the Dietary Approaches to Stop Hypertension (DASH) called MIND (Mediterranean-DASH Intervention for Neurodegenerative Delay), was devised in 2015 to emphasize the mitigation of cognitive decline [75, 76]. MIND promotes leafy green, berry, and poultry intake while minimizing the consumption of fried food and sweets. One recent study showed a uniquely protective effect for PD for the MIND diet beyond strict MeDi adherence, but only among women [77].

Preliminary work is underway to explore the mechanism of such benefit. Some researchers have used Mendelian Randomization to support a causal relationship between high dairy intake and higher PD risk [78], whereas others have looked at the inflammatory index of Western versus Mediterranean diets and shown that the former is more pro-inflammatory, and the consumers of such were much more likely to develop prodromal PD [79]. Adding to this, a small single-center randomized controlled trial of the MeDi in patients with PD did show that after ten weeks the serum total antioxidant capacity was significantly higher [80]. Explorations of the role of individual antioxidant micronutrients have yielded inconclusive results and have such wide-ranging effects, that it is more likely that the dietary milieu is beneficial rather than any individual antioxidant [81]. Yet others have implicated the gut microbiome, but it remains unclear if the MeDi promotes gut microbiome health and brain health via two separate mechanisms, or that gut biosis or PD determine dietary habits, or that PD causes gut dysbiosis [82]. No current studies have evaluated all three together.

In stark contrast to the MeDi, nascent research in nutritional ketosis, whereby restriction of protein and carbohydrates are limited to <30% of total energy expenditure, may offer some benefit in PD. However, the limited studies have been small, uncontrolled, and characterized by poor adherence to the strict regimen [83]. Further research is ongoing in this area.

While it is more likely the entire dietary milieu is what underscores symptomatology, a few select vitamins have undergone more rigorous analysis. Vitamin B12 deficiency can cause insidious cognitive and psychiatric disturbances (depression and paranoia), neuromuscular deficits (ataxia, neuropathy, and paresthesia), and autonomic dysfunction (hyposmia, postural hypotension, incontinence, and impotence), mimicking and exacerbating the features seen in PD [84]. In a de novo PD population, B12 deficiency was found in nearly 20% of the population, and this correlated with greater declines in mini-mental status exam (MMSE) and ambulatory capacity [85]. This is despite no differences detected in hematologic abnormalities. Vitamin D studies in PD patients have shown inconsistent results, but some studies have shown an inverse relationship with falls [86], cognition and mood [87], whereas interventional studies have been lacking [88]. At this point, vitamin D deficiency should be screened for and addressed according to major society guidelines. Research into other antioxidants such as vitamin C, resveratrol, curcumin, selenium and more are in their infancy [81].

One subfield of nutrition research with many unresolved questions pertains to the gut microbiome. The microbiome generally changes across the lifespan, and more dramatic changes occur in PD: many members of the enterobacteriacae species are increased whereas members of prevotella and lactobacillae are decreased [89]. It is thought that this dysbiosis leads to gut epithelial cell disruption, a release of bacterial toxins and antigens, activation of immune cells and release of pro-inflammatory cytokines which can cross a leaky blood-brain barrier and ultimately drive alpha-synuclein aggregation. Probiotics, live microorganisms available as over-the-counter capsules, may potentially support gut and overall host health through reducing intestinal permeability, microbial translocation, and neuroinflammation[90]. In the limited studies performed in the PD population, various probiotics have shown a significant improvement in constipation [91] and even in motor function and inflammatory markers [92]. It is likely the right admixture of probiotic strains will need to be personalized as the technology to do so becomes more readily available.

*Alcohol*. While several studies on alcohol consumption and incident PD risk have been published, there is a lack of uniformity on reporting alcohol content and frequency, making large-scale conclusions challenging. Underscoring this heterogeneity, the largest study performed on alcohol use indicated no significant association, but beverage-specific analysis indicated heavy liquor consumption was associated with an increased risk whereas infrequent beer consumption provided a protective effect [93]. Overall, when accounting for methodological differences and known confounders, alcohol does not appear to have a significant association with incident PD risk beyond its neuroprotective effect as included in the Mediterranean diet (see above) [94]. Nevertheless, it is well established that alcohol is linked to myriad health complications. More specifically, those with mild cognitive impairment, may be at greater risk for progression to dementia when consuming alcohol [95]. Therefore, abstinence might be recommended.

*Anticholinergic and other potentially detrimental medications*.

Anticholinergic burden is a concept used to quantify the cumulative effect of exposure to drugs with anticholinergic properties. Anticholinergic side effects include confusion, hallucinations, dry mouth, blurred vision, constipation, and urinary retention, with the elderly being particularly susceptible. In this geriatric population, anticholinergic exposure is strongly associated with hospitalization and mortality[96]. Specifically in PD, patients with higher anticholinergic burden are more likely to have fractures, delirium, and more frequent emergency department visits[97], more freezing of gait [98] and an increased risk of dementia[99], which is likely dose-dependent [100]. While the mechanism is unknown, it is possibly mediated through enhanced deposition of amyloid plaques[101]. Compounding this effect is evidence that PD patients exhibit impaired cytochrome P450 2D6 (CYP2D6) function, the key enzyme responsible for the metabolism of most anticholinergic drugs which contribute to increased adverse drug events [102]. Given all of this, one may consider calculating an anticholinergic burden using any of the several available tools (Anticholinergic Cognitive Burden scale (ACB) [103], Anticholinergic Drug Scale (ADS) [104] and the Anticholinergic Risk Scale (ARS) [105]) and de-prescribing as able. While it is uncertain if de-prescribing anticholinergics is protective against cognitive decline, a clinical trial investigating this question is ongoing [106]. A number of other potentially detrimental medications for the older population - including, but not limited to, dopamine-blocking anti-emetics and antipsychotics, benzodiazepines, central alpha-agonists, sedative-hypnotics, antihistamines (largely because of their anticholinergic side effects) and gabapentinoids - have been collated in a recent update to the American Geriatric Society’s Beers List [107].

## 3c. Modifiable Prodromal Symptoms

Prodromal symptoms refer to any feature that indicates some element of neurodegeneration is present; however, they do not imply that manifest PD can automatically be diagnosed. Table 2 describes a summary of known prodromal symptoms, with greater detail offered below.

*Depression*. The first epidemiological study to evaluate depression and incident PD was performed within a large network of primary care clinics in the Netherlands. At the time of diagnosis of PD, 9.2% had a history of depression whereas only 4.0% of a matched control population had such a history [113]. A larger, later study performed in the United States confirmed this finding [114]. This was again recapitulated in a Swedish population, whose authors showed a time-dependent effect[115]. Moreover it is unlikely that antidepressant medications are to blame, but rather it indicates a shared pathophysiology, that of a deficit in monoaminergic transmission [116].

*Neurotrauma.* Post-traumatic stress disorder (PTSD) and traumatic brain injury (TBI) are both emerging risk factors for PD. PTSD was first shown to be linked with greater odds of development of RBD [123] and later with risk of incident PD [124]. This risk factor is particularly relevant among the veteran population. One plausible mechanism which may explain the association is that chronic stress may drive pro-inflammatory cytokine secretion and accelerate neurodegeneration, particularly within the hippocampus and the amygdala [125]. Proponents of the brain-first hypothesis of PD point to evidence of early deposition of alpha-synuclein aggregates within the amygdala [126]; these individuals with accelerated aging of the area may be more susceptible to abnormal protein accumulation. TBI is likewise particularly important in the veteran population, where it has been shown that even those with just a mild TBI may have a 56% higher risk for developing PD later in life [127]. Similarly, neuroinflammation has been suggested as a potential mechanism which could nucleate or accelerate PD pathology[128]. Unpublished observations suggest there is also a synergistic effect on incidence of PD [129]. As inflammation may underlie both chronic TBI and PTSD, it is plausible that any treatments directed against them may change the milieu enough to alter the course of neurodegeneration.

*Emerging associations*. A large study of 1.5 million veterans with pathology specimens revealed several gastrointestinal (including gastroesophageal reflux), genitourinary, and skin disorders manifest decades before diagnosis of PD, with dermatophytosis and prostatic hyperplasia as novel high prevalence prodromal disorders [130]. The latter is of interest as preclinical and epidemiological work has suggested that terazosin, an α1-adrenergic receptor antagonist that is unique in its ability to stimulate phosphoglycerate kinase 1 - the first ATP-generating enzyme in glycolysis - via its quinazoline motif, , may slow neuronal death and delay PD progression [131]. Lastly, a nested case-control study of a diverse population of low socioeconomic status in London added to the literature by identifying epilepsy and hearing loss as novel risk factors for developing incident PD [132]. Further research is warranted in these areas to link them mechanistically to PD. A major limitation is that the authors could not adjust for anti-seizure medication types, many of which are well-known to mimic parkinsonian symptoms.

*Interaction of risk factors and prodromal symptoms.* Few studies have looked at the interaction of risk factors, rather researchers typically study them in isolation, with a few exceptions. Two studies have shown independent effects of tobacco and caffeine consumption on PD incidence, without any clear evidence for synergy [22, 65]. One published study does report synergistic effects of vascular risk factors on cognitive outcomes [43] and some unpublished observations do suggest there is a synergistic effect on incidence of PD between TBI and PTSD [129]. It is conceivable, in fact likely, that other interactions exist, but this work has yet to be explored in great detail in PD.

## 3d. Future Directions: Using Biomarkers to Personalize Prevention

Several candidate markers with pathophysiologically-plausible, and compelling, evidence have been published recently (table 3). At present it is unclear how best to incorporate them in clinical practice because of the lack of prospective, long-term studies.

*Neuroimaging*. Since neuronal degeneration in the substantia nigra pars compacta (SNpc) is the key pathologic hallmark, attempts have been made to measure this non-invasively. The area of greatest interest are the dopaminergic neurons within nigrosome 1 (N1). The N1 subregion can be visualized using magnetic resonance imaging (MRI), taking advantage of the contrast between differences in iron concentration and/or oxidative state in N1 and the rest of SNpc. In patients with PD, N1 signal is lost, compared to healthy controls [133-136], but it is currently under investigation in the prodromal phase. A similar technique exploiting the fact that neuromelanin pigment is paramagnetic has shown robust differences in the PD population compared to controls, and is also under investigation in the prodromal phase [137]. An alternative approach to structural evaluation has explored measurement of free water diffusion within the SNpc, where there is a significant elevation of free water compared to controls and that this continues to increase with disease progression [138]. No studies have yet explored this in the prodromal period. Yet another approach to structural analysis is the use of transcranial ultrasound (TCS). Hyperechogenicity in the area of the midbrain has been consistently found in 79 to 90% of patients with PD using US [139, 140]. However, up to 13% of these studies provide inconclusive results and another 12% of healthy controls exhibit hyper-echogenicity [141]. The false positive rate is even higher in patients with essential tremor [142].

Other imaging tools are available to evaluate neuronal function. The most widely-used clinically, is the DAT SPECT scan. Even those with preclinical parkinsonism, such as hyposmic relatives of PD patients [143] or those with RBD [144] can demonstrate an abnormal DAT SPECT, but the specificity is poor. A further limitation is that it cannot distinguish among the other neurodegenerative causes of parkinsonism. Lastly, recognizing that brain mitochondrial dysfunction is an early and consistent hallmark of PD, it may prove prudent to measure mitochondrial function and brain energetics *in vivo*. Magnetic resonance spectroscopic imaging (MRSI) pairs the spatial mapping of conventional MRI with the targeting of radio frequencies to excite specific target regions to obtain a profile of phosphate-containing molecules [145]. The phosphate-containing molecules measured using this technique include many related to mitochondrial function; adenosine triphosphate (ATP) is the major source of cellular energy, its breakdown products adenosine diphosphate (ADP) and free, inorganic phosphate (Pi), and phosphocreatine (PCr), an energy store allowing fast regeneration of ATP in response to high metabolic demand. While use of this technique has been limited in the PD population, several authors have detected an impairment in the balance of ATP synthesis and breakdown, indicating failure to adequately keep up with metabolic demands [146-149].

Separate from measures of neuronal integrity and function are emerging markers of glymphatic function. Perivascular spaces (PVS) are fluid-filled cavities that surround perforating vessels in the brain. CSF flows into the brain interstitium from arterial PVS and then is directed outward through the venous PVS resulting in clearance of metabolic waste. Glymphatic clearance appears to be sleep-dependent as studies have shown enhanced flux during non-REM sleep [150]; conversely, sleep disturbances drive enhanced abnormal protein accumulation [151]. These PVS can be detected on MRI. While simple quantitation has shown they are typically associated with the elderly and those with vascular comorbidities - and likewise are directly associated with greater cognitive dysfunction [152] - diffusion tensor image analysis along the perivascular space (DTI-ALPS) has emerged as a probable better measure of glymphatic function [153]. Higher DTI-ALPS index indicates better glymphatic function. Research in PD specifically is in its infancy. However, early studies have shown that there is impaired glymphatic function in PD patients when compared to healthy controls[154], when compared to essential tremor [155], when compared over time with accumulating cognitive dysfunction[156], and even when comparing the prodromal RBD population to healthy controls [157]. While research continues to refine the techniques, perhaps the most important takeaway is that addressing sleep-active glymphatic function could have a direct benefit.

*Serologic markers*. Several serologic markers are under investigation, but two have gained widespread interest. Neurofilaments are abundant structural scaffolding proteins exclusively expressed in neurons, and while their precise function remains unknown, their detection is highly specific to neuronal damage. While first investigated in CSF, it has since been shown that elevated levels can be detected in serum in PD compared to controls[158] and may correlate with disease severity and progression[159]. A second marker, nuclear factor erythroid 2-related factor 2 (NRF2), a master regulator of the antioxidant response, is also under investigation. NRF2 has been proposed to play a role in PD biology as genetic haplotypes which result in increased NRF2 transcription are associated with delayed onset and overall decreased risk of PD [160]. Elevated transcript and protein levels have been detected in PD patients, compared to healthy controls, and there may also be an effect based on disease severity [161]. While not specific to PD [162], further work is ongoing as drugs targeting this pathway hold promise [163].

*Real-time Quaking-induced Conversion (RT-QuIC)*. Since manifold non-motor symptoms manifest in PD, alpha-synuclein has been hypothesized to be broadly distributed in peripheral tissues. In fact, it can be reliably detected throughout the integument via immunohistochemistry with a simple punch biopsy [164, 165]. More recently, Donadio and colleagues [166] showed through RT-QuIC that this measured alpha-synuclein has impressive aggregation ability and can discriminate between PD and controls and from other non-synucleinopathy neurodegenerative disorders. It also compares favorably with RT-QuIC undertaken in CSF. Early multi-center studies have shown the ability to detect alpha-synuclein in peripheral tissues [167] and aggregating ability via RT-QuIC in CSF [168] in RBD cohorts.

*Genetic and epigenetic risk*. While rare high-penetrance genetic mutations have a correspondingly high risk of incident PD, the cumulative predictive effect of common and low individual effect strength genetic risk variants have also been explored. In the most recent meta-analysis of genome-wide association studies [169], 90 risk loci have been identified, and those with the highest quartile of polygenic risk exhibit a 3.51-fold increased risk of incident PD compared with those in the lowest quartile. The same has been shown in one Greek population for prodromal PD [170]. In contrast, epigenetics is the study of reversible heritable changes in gene expression, such as DNA methylation, chromatin remodeling and microRNA (miRNA) regulation, that occur without alterations to the DNA sequence itself, thus linking the genome with the environment [171]. Early studies have shown that epigenetic changes accumulate with aging and can reliably discriminate between neurodegenerative disease and healthy, age-matched controls, but further studies are necessary to make this more useful [172].

*Integration of biomarkers*. It is likely that a combination of biomarkers can improve prediction of phenoconversion to frank PD, but few studies have exploited this approach. For example, Jennings et al. have shown that the combination of abnormal olfactory testing and abnormal DAT SPECT imaging increases the relative risk of developing PD by 17-fold over and above imaging alone [173]. Persistent hyposmia on repeated testing further increases this risk [174]. The same was shown for combining hyposmia with hyperechogenicity or with positive family history. In fact, either combination was able to predict phenoconversion in these patients at 5 years prior to formal diagnosis, something any single marker alone could not do [175]. Combining multiple imaging modalities together is enticing as they can often be collected together. In fact, various pairings of N1 imaging, neuromelanin-sensitive scans, DAT SPECT, and DTI scans have shown improved diagnostic accuracy in a cohort of probable PD over any single study [176, 177]. The combination approach in the prodromal population is more limited. In an RBD population, combined studies improve certainty of nigral damage, and improve discrimination of RBD from healthy controls, but have not yet been shown to predict incident PD [178], with the exception of Iranzo and colleagues who have combined DAT SPECT with transcranial ultrasound to improve the ability to predict phenoconversion in an RBD population [144]. Future work combining serologic, genetic, and imaging markers would therefore be a welcome addition.

## 3e. Important Negative Studies

Since patients at risk of neurodegeneration may seek out alternative treatments that could be available for purchase over the counter, it is important to review some of the recent landmark studies which have failed to show a beneficial effect in PD progression to avoid unnecessary exposure to side effects or sunk costs.

Strong evidence suggests that mitochondrial dysfunction and increased oxidative stress play a pivotal role in the pathogenesis of PD. The earliest study approaching this underlying mechanism evaluated 2000 IU per day of vitamin E with or without selegiline, and showed no benefit on disease progression or levodopa-associated complications [179]. More recently, multiplex assays from preserved plasma samples from this study may have identified a subgroup likely to respond to vitamin E, but this personalized medicine approach is not yet ready for widespread clinical application [180]. Vitamin E is considered relatively benign, but in excessive doses, it may inhibit vitamin K-dependent enzymes and increase risk for bleeding.

The next compound derived along this line of thought was Coenzyme Q10 (CoQ10), a key component of the electron transport chain leading directly to the generation of ATP, and more indirectly to reducing reactive oxygen species[181]. It also had the benefit of lacking significant side effects, even at extreme doses [182]. While a smaller phase II study suggested a trend toward a decrease in functional decline over 16 months, the definitive phase III double-blind RCT showed no evidence for benefit [183]. Mitoquinone (MitoQ), a more lipophilic antioxidant that accumulates within mitochondria several-fold greater than CoQ10, showed no benefit in disease progression over placebo at 12 months of median follow-up [184].

Glutathione, another antioxidant known to be significantly reduced in the brain of PD patients, and considered ‘generally recognized as safe’ by the FDA, also failed to show efficacy in a 12-week phase II study evaluating thrice-weekly dosing of intravenous glutathione compared to placebo [185]. While there may be a beneficial effect if the problem of blood-brain barrier permeability could be overcome, we must agree with other pre-eminent leaders in the field who suggest “patients with PD should be encouraged to say no to an IV placed in their arm for the false hope of a symptomatic glutathione treatment” [186]. Creatine, another compound proposed to improve mitochondrial bioenergetics, emerged later from a group of four candidate drugs to delay disease progression based on non-futility analyses to a full phase III RCT. While the drug was well-tolerated, the trial was ultimately stopped for futility after a planned interim analysis of 4 years median follow up [187]. Finally, urate, an end-product of purine metabolism, serves as a circulating antioxidant, and while excessive levels can cause crystallopathic disorders such as gout or nephrolithiasis, elevated levels in healthy individuals have been shown to be protective against incident PD [188]. However, an interventional study evaluating the urate-elevating inosine, showed no improvement [189]. Current genomic analysis is underway to determine if some sub-populations may respond.

## 4. Considerations for Referral to a Preventive Clinic and Approach to Counseling

**Figure 2.**
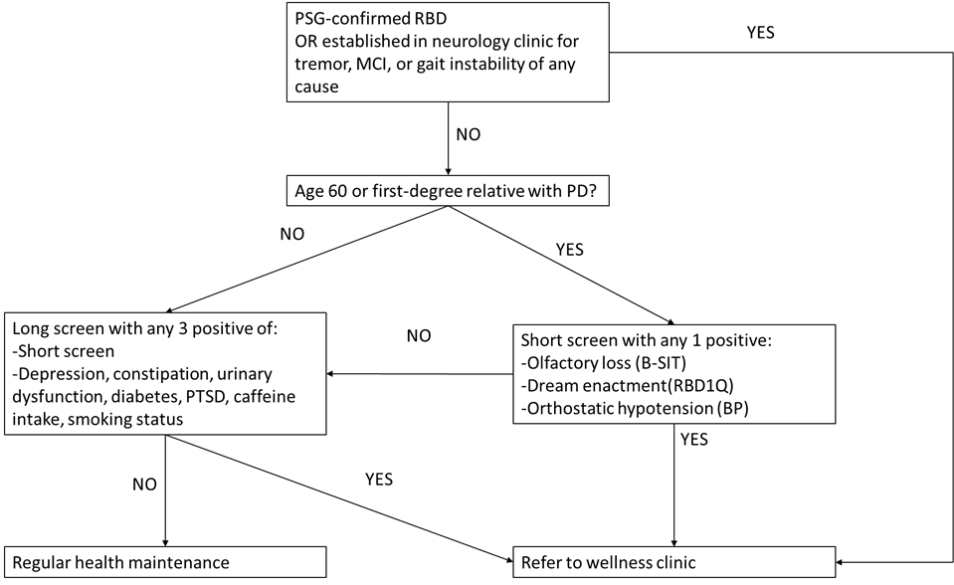
**Flow diagram depicting proposed method of referral to wellness clinic for patients at risk**. Automatic referrals would come through sleep medicine or neurology clinics. The next group would come from geriatrics or internal medicine clinics following a short or long screen. PSG: polysomnogram; RBD: REM Behavior Disorder; MCI: mild cognitive impairment; B-SIT: Brief smell identification test; RBD1Q: RBD single question screen; BP: blood pressure; PTSD: Post-traumatic Stress Disorder. The wellness clinic practitioners can then create a personalized intervention strategy based on tables 1 and 2.

It would be imprudent to permit unchecked referrals to a preventive clinic given the aging population and resource scarcity. Therefore, we have devised a flow chart outlining the process for referral screening based on a tiered system of risk (Fig. 2).

**Table 1:** Potentially modifiable risk factors.

Risk Factor	Adverse Outcome	Screening Strategy	Optimization Strategy
**Smoking**	Stroke Cognitive decline	History	Should be counseled to quit due to deleterious effects in other body systems Consider nicotine patch Consider greater consumption of nicotine-containing foods (e.g. tomatoes, eggplants, potatoes, peppers)
**Diabetes mellitus, type 2**	PD Incidence Gait dysfunction Cognitive decline	HbA1C	Engage in diabetes clinic Pharmacotherapy as needed to maintain euglycemia, consider GLP1 agonist if appropriate MeDi
**Hypertension**	Stroke Cognitive decline	BP screening	Treat according to established guidelines Consider avoiding propranolol. If using for tremor, consider closer follow up Consider using CCBs
**Hypercholesterolemia**	PD Incidence Cognitive decline Stroke	Lipid panel	Treat according to established guidelines Encourage adherence to statin therapy, if appropriate MeDi
**Physical inactivity**	PD Incidence Motor symptom progression Cognitive decline	History	Recommend 30min exercise, 5 days weekly High intensity (80-85% maximum HR) is possibly more beneficial Incorporate progressive resistance training and tai chi
**Caffeine**	PD Incidence	History	Consider regular caffeinated coffee or tea intake (minimum 300mg caffeine weekly)
**Western diet**	PD Incidence	FFQ	Maximize vegetables, legumes, fruits and nuts, cereal, fish, polyunsaturated fats Minimize saturated fats, meat, poultry, excess sugar, and dairy products Consider moderate alcohol intake Consider regular probiotic supplementation
**Micronutrient deficiency**	Cognitive decline Gait disturbance	Serum vitamin B12, homocysteine Serum vitamin D Serum vitamin E	MeDi Replete according to guidelines Consider higher targets for B12 (<234 pmol/L) and Homocysteine (>11 umol/L) Vitamin E target unknown
**Alcohol**	Cognitive decline Gait imbalance	History	Encourage abstinence or consider moderate intake consistent with MeDi
**Anticholinergic burden**	Cognitive decline Gait dysfunction	ACB ADS ARS	De-prescribing

These risk factors are associated with PD incidence or symptom progression. Each risk factor has a suggested screening strategy and recommendations for intervention. HbA1c: glycated hemoglobin; GLP1: glucagon-like peptide 1; MeDi: Mediterranean diet; BP: blood pressure; CCB: calcium channel blockers; FFQ: food frequency questionnaire; ACB: Anticholinergic Cognitive Burden scale; ADS: Anticholinergic Drug Scale; ARS: Anticholinergic Risk Scale.

RBD, the combination of documented REM sleep without atonia (RSWA) and a history of dream enactment behavior, is the single greatest predictor of later development of synucleinopathy [14]. With five pooled prospective studies yielding a 75% phenoconversion rate, this equates to a positive likelihood ratio of 130. Of course, it is important to be certain it is idiopathic, and not confounded by medications, alcohol, or other conditions. Further risk stratification can be done based on age or other risk factors [190], but is otherwise unnecessary for this purpose. Sleep medicine physicians will therefore serve as a powerful referral source as well as education and increasing awareness among general practitioners.

Neurology clinics will also serve as an important referral source. They are often tasked with diagnosing and managing tremors, gait difficulties and cognitive impairment. Healthcare records studies indicate that motor impairments may start 6-10 years prior to the formal diagnosis of PD, with the most common findings being tremor and imbalance [191]. This was recapitulated in a prospective population cohort study, where bradykinetic features and tremor preceded diagnosis of PD by 6 years [192]. Essential tremor deserves special mention since there is considerable debate over to what extent subtle parkinsonian features are acceptable in this diagnosis, or if co-pathology is truly present [193]. Several population studies have shown that PD is of much higher prevalence within the ET population compared to healthy controls [194, 195] and even when compared against a population with other movement disorders [196]. Considering the prominent role of beta-blockers, specifically propranolol in this population [197], which may accelerate or contribute to parkinsonism, this population is worth monitoring closely. The last cohort within the neurology clinic worth close scrutiny is those with cognitive impairment. Cognitive deficits have been shown to be associated with increased PD risk in 2 prospective studies investigating global cognition [198] and cognitive decline [199]. In fact, the presence of cognitive impairment yields a 2-fold increased risk of subsequent PD.

Beyond these considerations of an ‘automatic’ referral, other features are worth attention. The next most powerful risk factor for PD is age, with the prevalence starting at 0.4% for those aged 50 and jumping to 4% for those over age 80. We therefore recommend evaluating those at age 60, where the prevalence rises to 1.25% or those with a family history of PD based on the likelihood that polygenic risk score may be high. From here, a short-screen can be undertaken which is simple to administer and evaluates high-risk features such as hyposmia, orthostatic hypotension, and RBD (utilizing the RBD1Q which asks for a simple yes/no response to the question: “Have you ever been told, or suspected yourself, that you seem to ‘act out your dreams’ while asleep (for example, punching, flailing your arms in the air, making running movements, etc.)?” [200]). Each of these increases the risk of developing PD 3- to 6-fold.

Any other individual can also undergo a longer screening process and be considered for referral. Those who screen negative are unlikely to be in the prodromal phase and should continue with their regular health maintenance. Those who screen positive should be considered for referral.

**Table 2:** Potentially modifiable prodromal features.

Prodromal Symptom	Adverse Outcome	Screening Strategy	Optimization Strategy
**Constipation**	PD incidence Cognitive decline	History	MeDi Consider probiotic supplementation Regular exercise Polyethylene glycol if needed Avoid dopamine-blocking gastroprokinetic medications, e.g. metoclopramide
**Depression**	PD Incidence	BDI GDS	Treat with medications as appropriate Consider referral to CBT to build coping strategies Be aware bupropion can interfere with dopamine transporter scans
**TBI**	PD Incidence	HTEC	Treat according to guidelines, referral to polytrauma clinic
**PTSD**	PD Incidence	PCL-5 CAPS-5	Consider mental health referral for diagnosis and treatment
**Urinary dysfunction**	PD Incidence Restriction of engagement with social and physical activities	History	If BPH is primary problem, consider treatment with terazosin if indicated Minimize anticholinergic medications
**RBD**	PD Incidence Motor and Non-Motor symptom severity	RBD1Q Polysomnogram	Annual screen for movement abnormalities, lower threshold for referral to movement disorders Consider melatonin If available, prodromal clinical trial referral
**Hyposmia**	PD Incidence	B-SIT	Annual screen for movement abnormalities, lower threshold for referral to movement disorders
**Orthostatic hypotension**	PD Incidence	Orthostatic vitals measurement	Optimize BP to permit regular exercise participation Lower threshold for referral to movement disorders
**Motor impairments**	PD Incidence	Neurological exam	Annual screen for movement abnormalities, lower threshold for referral to movement disorders

These prodromal features are associated with PD incidence or symptom progression. Each risk factor has a suggested screening strategy and recommendations for intervention. MeDi: Mediterranean Diet; BDI: Beck Depression Inventory; GDS: Geriatric Depression Scale; CBT: Cognitive Behavioral Therapy; TBI: Traumatic Brain Injury; HTEC: Head Trauma Events Characteristics; PTSD: Post-traumatic Stress Disorder; PCL-5: PTSD Checklist for DSM 5; CAPS-5: Clinician Administered PTSD Scale for DSM-5; RBD1Q: REM Behavior Disorder Single Question Screen; B-SIT: Basic Smell Identification Test

**Table 3:** Summary of biomarkers under investigation for prodromal PD.

Biomarker	Brief description of measure
**MRI**	Evaluates differences in tissue contrast, neuromelanin, free water diffusion within the substantia nigra
**Transcranial Ultrasound**	Evaluates differences in hyperechogenicity within the midbrain
**DAT SPECT**	Evaluates differences in dopamine transporter tracer uptake within the striatum
**Phosphorus MRSI**	Evaluates mitochondrial bioenergetics within the brain
**DTI-ALPS**	Evaluates glymphatic function within the brain
**Serological markers**	Several non-specific markers of neuronal damage or antioxidant response can be measured. No consistent marker, or panel of markers, is routinely used yet in clinical practice
**RT-QuIC**	Alpha-synuclein aggregation can be detected in serum and CSF samples
**Genetic and epigenetic markers**	Several emerging genetic risk loci and epigenetic modifications may predispose to progression to frank PD

MRI: Magnetic Resonance Imaging. DAT SPECT: Dopamine Transporter Single-photon emission computed tomography. MRSI: Magnetic Resonance Spectroscopic Imaging. DTI-ALPS: Diffusion Tensor Imaging Analysis along the Perivascular Space. RT-QuIC: Real-time Quaking-induced Conversion.

Once in the clinic, we propose a thorough intake of the aforementioned risk factors and prodromal features, as well as a full neurological exam to identify overt parkinsonism or more subtle findings that are worth monitoring. If imaging is available, this is also reviewed. This data is then synthesized to determine where on the PARS pyramid the patient may fall, which can be defined quantitatively using prodromal calculators proposed by the Movement Disorder Society [7], using 80% as a cutoff for defining prodromal PD.

Using the available evidence, we propose to personalize a multi-modal approach for intervention designed around the extant risk factors (Table 1) and prodromal symptoms (Table 2) after completing a detailed inventory. We first recommend vascular risk factor modification: quitting smoking, managing diabetes mellitus, controlling blood pressure, and controlling high cholesterol. We next counsel on the importance of physical activity. This includes a balance of high-intensity aerobic exercise for potential disease modification, along with resistance training and balance training to improve balance. We next counsel on the potential benefits of the Mediterranean diet, supplementation with vitamins D, B12, and E if appropriate, and suggest incorporating daily caffeine intake. Lastly, we take a detailed review of their medication list and ensure each one has a clear and beneficial purpose, with particular attention paid to anticholinergic medications.

After this, we turn our attention to the prodromal symptoms. While many of the above interventions can address these, too, some targeted therapies are available. For constipation, we consider adding medications such as polyethylene glycol or probiotics if dietary modifications are insufficient. For depression and neurotrauma, we refer our patients to mental health professionals. For urinary dysfunction, we suggest urology referral or consider use of terazosin. For RBD, we would confirm with PSG and treat with melatonin. More troublesome cases can be referred to sleep specialists. These recommendations do overlap with the aforementioned proposal of 12 risk factors which have been shown to prevent or delay onset of ADRD [9]. Commonalities include treating diabetes, obesity, high blood pressure, depression, encouraging smoking cessation and frequent exercise, and avoiding excessive alcohol intake. While PD and ADRD are distinct pathologies, this overlap likely supports the disease-delaying potential of the recommendations proposed herein. t

## 5. Conclusions

Herein we describe the growing epidemic of PD and the ever-growing group of individuals at risk for developing it and other neurodegenerative synucleinopathies. While some prodromal symptoms substantially increase the risk of incident PD, others do so only marginally. We propose a framework for identifying this population, for stratifying their risk, and we offer several tools for consideration to support their health. In contrast to typical one-step interventions, we propose a multimodal approach, recognizing that adherence to all will be limited and that there is some power in combining strategies. At this time, it is unknown the optimal time to implement these strategies, but it is likely most efficacious in the pre-clinical phase. This review represents recommendations based on expert opinion; further rigorous prospective studies are warranted.
